# Changes in muscle coordination patterns during 400‐m sprint: Impact of fatigue and performance decline

**DOI:** 10.1002/ejsc.12085

**Published:** 2024-02-17

**Authors:** G. Kakehata, H. Saito, N. Takei, H. Yokoyama, K. Nakazawa

**Affiliations:** ^1^ Department of Life Sciences Graduate School of Arts and Sciences The University of Tokyo Tokyo Japan; ^2^ Faculty of Sport Sciences Waseda University Saitama Japan; ^3^ National Youth Sports Institute Singapore Singapore; ^4^ Department of Physical Therapy Tokyo University of Technology Tokyo Japan; ^5^ Institute of Engineering Tokyo University of Agriculture and Technology Tokyo Japan

**Keywords:** biomechanics, fatigue, performance

## Abstract

The purpose of this study was to compare the muscle synergies extracted from 14 unilateral lower‐limb and trunk muscles between the first and final parts of a 400‐m sprint in experienced sprinters to understand neuromuscular coordination of multiple muscles in the fatigued condition sprint. Nine male 400‐m sprinters (400‐m personal record: 48.11 ± 1.6 s) performed 400‐m sprints as with the real competition strategy. We defined the first part (100–150 m section) and the final part (350–400 m section), and obtained mean spatiotemporal variables (e.g., running speed, step frequency, and step length) for both parts. Electromyography (EMG) signals were obtained using wireless EMG sensors (2000 Hz) from 14 lower‐limb and trunk muscles. Non‐negative matrix factorization was performed to extract the muscle synergies for both parts. We observed significantly declined spatiotemporal variables in the final part induced by fatigue. The extracted number of synergies was 7.0 ± 0.7 (mean ± SD) for the first part and 7.2 ± 0.4 for the final part with no significant differences between parts. However, we identified specific muscle synergy, and alterations in the individual muscle weightings of several hip muscles (rectus femoris: RF, tensor fasciae latae: TFL, and glutes maximus: Gmax muscles) while there was no change in the muscle weighting of shank muscles and the temporal patterns of all muscles even following fatigue in the 400‐m sprint. Fatigue‐induced performance decline in a 400‐m sprint corresponds to alterations in muscle synergies, particularly in hip muscles, with notable shifts in RF, TFL, and Gmax activation.

## Highlights

- Running speed, step frequency, and step length significantly declined in the final part (350–400 m) compared to the first part (100–150 m) during the 400‐m maximal sprint due to fatigue.
- Number of muscle synergies was no significant differences between the first part (7.0 ± 0.7) and the final part (7.2 ± 0.4).
- Several hip muscle weighting components were altered, while no changes were observed in the shank muscles. The temporal patterns of all muscles remained consistent even after fatigue in the 400‐m sprint.

## 1 INTRODUCTION

The 400‐meter sprint (400‐m) is the longest sprint event in athletics. The 400‐m athletes try to optimize their combination of step frequency and step length to maintain running speed as much as possible in the final part (Hanon et al., 2009). However, both components are much decreased in the final part, therefore, deceleration is more dramatic than other sprint events (Casado et al., 2021). One major factor of deceleration in the 400‐m would be explained from physiological aspects. Overall, energy production from the aerobic‐anaerobic system drastically decreased in the final part of the 400‐m (Spencer et al., 2001), and then athletes are forced to extreme fatigue. Thus, the muscle fatigue in the 400‐m has been well analyzed from the physiological aspects such as the cytosolic glycolysis, and aerobic‐anaerobic mechanisms as well (Hirvonen et al., 1992; Nummela et al., 1992). In parallel with physiological mechanisms, the running biomechanical mechanics is also one of the limiting factors of the 400‐m. For instance, knee flexion and extension movements during ground contact are much larger in the final part of the 400‐m (Saraslanidis et al., 2011), which results in increasing ground contact time. In addition, the leg stiffness in the final part of the 400‐m is a 40% decrease compared to the maximal speed phase (Hobara et al., 2010). In consequence, athletes are unable to maintain the force production capability (i.e., decreases ground reaction forces) in the final part of the 400‐m (Nummela et al., 1994), which induces a greater decrease in step length.

In addition, previous studies have analyzed the lower‐limb muscle activity amplitudes using electromyography (EMG) signals during 400‐m (Nummela et al., 1992, 1994). Nummela, Vuorimaa (Nummela et al., 1992) demonstrated that maximal EMG amplitudes of the gastrocnemius (GAS) and vastus lateralis (VL) muscles were more recruited in the final part of the 400‐m. Similarly, averaged EMG activity of GAS, VL, biceps femoris (BF), and rectus femoris (RF) in the final part of 400‐m was significantly higher than unfatigued sub‐maximal speed running (Nummela et al., 1994). Increasing the EMG amplitude level during the fatigue condition is considered a struggle with depression of performance. Furthermore, the activation timing of the RF and BF muscle were significantly delayed in the deceleration phase than maximal speed phase in the 100‐m dash (Kakehata et al., 2022). Thus, athletes are not able to maintain muscle activation timings even in the shortest sprint event and changes in these thigh muscle activation timings are one of the limiting factors of the 100‐m performance. These findings suggest that thigh muscle activation levels and activation timings would not be identical between the unfatigued and the fatigue condition sprint. However, these previous insights are based on analyzing individual muscles. Thus, it remains unclear how individual muscles interact or coordinate with multiple muscles in fatigue condition sprint.

It is important to note that the influence of muscle activities on motor function cannot be isolated to singular muscle groups (Chiel et al., 2009; Dickinson et al., 2000; Hooper et al., 2000). Motor function necessitates the harmonious orchestration of numerous muscles throughout the body. Thus, to gain a more comprehensive understanding of the mechanics of under‐fatigued sprint running, analyses involving multiple muscles are required. In this context, there has been the premise that the central nervous system relies on an efficient way to choose the control signal from a wide subspace by using a finite set of motor modules or muscle synergies formed by a flexible combination of muscle activation (d'Avella et al., 2003). Dimensionality reduction algorithms such as non‐negative matrix factorization (NMF), allows us to identify the minimum number of muscle synergies necessary to the execution of a task, their activation over time, and the contributions of muscles to each synergy (Ting et al., 2005; Torres‐Oviedo et al., 2007). Thus, muscle synergies can be correlated with the mechanical output of the human movement system (Ting et al., 2007, 2015), and have been previously suggested for the evaluation of running performances under different conditions (Cheung et al., 2020; Saito et al., 2018; Yokoyama et al., 2016).

Recent studies show that muscular fatigue can acutely alter muscle synergies, leading non‐fatigued muscles to adapt and compensate for the reduced or eliminated role of the fatigued ones (Ortega‐Auriol et al., 2018; Smale et al., 2016; Turpin et al., 2011). For example, Smale et al. noted a shift from a hip flexor to a hip extensor synergy after fatigue during single‐leg squats (Smale et al., 2016). Further, thigh muscle coordination is one of the limiting factors of the 100‐m dash performances (Kakehata et al., 2022). However, the neuromuscular compensation strategies of 400‐m sprinters in response to fatigue are currently unknown. This study, therefore, aims to compare the muscle synergies extracted from 14 unilateral lower‐limb and trunk muscles between the first and final parts of a 400‐m sprint in experienced sprinters. We hypothesized that disruptions in the neuromuscular coordination of multiple muscles (i.e., activation level and activation timing), especially thigh muscle, would be observed with performance decline for the 400‐m sprints. We believe that defining muscle synergies during the 400‐m sprint, and the effect of fatigue on sprint performance, will facilitate the development of effective strategies for high‐speed sprinting.

## 2 METHOD

### 2.1 Participants

Nine male Japanese 400‐m athletes (height: 1.78 ± 0.05 m, body mass: 68.3 ± 4.4 kg, age: 21.2 ± 1.8 years) volunteered to participate in the study. Their personal record of 400‐m was 48.11 ± 1.6 s and season best were 48.46 ± 2.0 s, respectively. This study was approved by the Ethics Committees. All subjects were informed of potential risks associated with the experimental procedures. Before the experiments, all subjects gave their written informed consent. All experiments were conducted in accordance with the Declaration of Helsinki.

### 2.2 Data acquire

After a self‐selected warm‐up activity (e.g., dynamic stretching, specific sprint drills, slow jogging to high‐speed sprinting), subjects performed a 400‐m sprint from a crouching start with a starting block (NF155B, NISHI) in the official 400‐m track in a lane five as is the real competition strategy (i.e., race pace). Then, all subjects used their own without carbon plate spike shoes. Each subject started to run when signaled by the sound of a track and field starting pistol (NG5085B, NISHI). Surface muscle EMG data were sampled at 2000 Hz using wireless EMG sensors (Trigno™ Wireless Sensor, Delsys). A portable wireless data logger (*H* = 12.3 cm, *D* = 2.0 cm, *W* = 6.8 cm, mass = 188 g) (Trigno™ Personal Monitor, Delsys) was attached to the subject's lower back to record EMG (Kakehata et al., 2022). EMG data were recorded from the 14 lower‐limb and trunk muscles (RF, BF, VL, vastus medialis: VM, gluteus maximus: Gmax, gluteus medius: Gmed, tensor fasciae latae: TFL, tibial anterior: TA, soleus: SOL, lateral head of GL, peroneus longus: RERO, rectus abdominis: RAS, external oblique: EO, erector spinae at L3: ESL3) from right side.

Before the sensors were attached, the involved area of skin was shaved and treated with alcohol to reduce inter‐electrode impedance. EMG signals for all muscles were checked after placing the loggers. In order to eliminate the influence of motion artifacts as much as possible, they were fixed with surgical tape and underwrap tape. We used one panning high‐speed camera (LUMIX DMC FZ‐300, Panasonic) to determine the moments of foot strike (FS) and foot off (FO) from the side of the running track at 240 Hz (Kakehata et al., 2021, 2022, 2023). Before starting a trial, in order to synchronize the FS and FO timing with the EMG data, the flash of the wireless all‐around light presenter (Synchronizer, Q'sfix) was recorded with the high‐speed cameras. The EMG data and the signal of the synchronizing device were fed into a software analysis program (EMGworks®, Delsys) via a synchronized sensor (Trigno™ Trigger Adapter) (Kakehata et al., 2022).

## 3 DATA PROCESSING OF SPATIOTEMPORAL VARIABLES

### 3.1 Kinematic variables

Reference markers were set every 50 m. The length of time it took for a subject's torso to travel between one reference marker and the next marker 50 m away was defined as the split time (sec). Then, running speed (m·s^−1^) was calculated by dividing 50 m by the split time. In addition, the contact time (ms) and flight time (ms) for each step were calculated from the number of frames of the high‐speed cameras, and the step frequency (Hz) was calculated for each step and then averaged. The step length (m) was calculated by dividing the running speed by the step frequency. Previous study demonstrated that the maximal speed reached the 50–100 m section in 400‐m, however, since this section is a curved lane it must have affected muscle activity. Thus, we defined the 100–150 m section as the first part (maximal speed limit) and the 350–400 m section as the final part (fatigue resistance limit) and obtained mean spatiotemporal variables for the two parts (First vs. Final).

### 3.2 Data processing of EMG

The raw EMG signals were filtered at 30 Hz using a fourth‐order Butterworth filter to eliminate motion artifacts (Redfern et al., 1993). Next, the signals were demeaned, full‐wave‐rectified, and filtered at 20 Hz using a fourth‐order Butterworth filter (Turpin et al., 2021). To ensure that each trial contributed equally to the extracted muscle synergies, the smoothed EMG envelopes were time‐interpolated with linear interpolation to generate 200 time points between the start and end points for each trial. To accurately extract muscle synergy, it is recommended to obtain the data variability in terms of muscle activations (Oliveira et al., 2014; Steele et al., 2015). Therefore, we created concatenated EMG matrices from 10 gait cycles for each phase for each subject. We defined one gait cycle as the time from the moment of foot‐strike of the right leg until the next foot‐strike of the right leg again. The EMG recording from each muscle was normalized to the maximum amplitude across phases. Each muscle vector in the data matrix was then standardized to have unit variance to ensure that the activity in each muscle was equally weighted.

### 3.3 Muscle synergy extraction

NMF based on the multiplicative update rules was applied to the all‐task EMG matrix to extract the muscle synergies (Lee et al., 1999; Tresch et al., 2006). NMF has previously been described as a linear decomposition technique (Lee et al., 1999; Tresch et al., 2006) according to Equation (1):

(1) M=W·C+e

where *M* (*m*  ×  *t* matrix, where *m* is the number of muscles and *t* is the number of samples, [ i.e., spatiotemporal profiles of muscle activity]) is a linear combination of muscle synergy weighting components, *W* (*m*  ×  *n* matrix, where *n* is the number of muscle synergies), temporal pattern components *C* (*n*  ×  *t* matrix, representing temporal patterns), and *e* is the residual error matrix. The initialization of *W* and *C* was set randomly (Lee et al., 1999; Tresch et al., 2006). We applied NMF to extract each possible *n* value from 1 to 14 from each dataset. To estimate the optimal number of muscle synergies, the variance accounted for (VAF) by the reconstructed EMG (M) was calculated at each iteration (Torres‐Oviedo et al., 2006). The VAF was defined as 100 × the square of the uncentered Pearson's correlation coefficient (Torres‐Oviedo et al., 2006; Zar, 1999). Considering the local minima inherent in NMF, each synergy extraction was repeated 50 times, and the VAF was calculated for each possible number of synergies. Considering the local minima inherent in NMF, each synergy extraction was repeated 300 times, and the VAF was calculated at each iteration. The iterations with the highest VAF were maintained (Saito et al., 2021, 2022, 2023; Yokoyama et al., 2016, 2021). VAFs >0.90% were used to identify the optimal number of synergies commonly used in the literature (Saito et al., 2021, 2022, 2023; Yokoyama et al., 2016, 2021). It was suggested that the criterion VAF >0.90% ensures a sufficient representation of the data (Turpin et al., 2021), although this is still debated (Barradas et al., 2020).

### 3.4 Clustering muscle synergy

We identified the representative synergy vectors across participants using hierarchical clustering analysis of muscle synergies for each part (Delis et al., 2018; Yokoyama et al., 2016). This clustering was performed using the MATLAB “linkage” function (Ward's method, Euclidean distance). The optimal number of clusters was determined using the gap statistic using the MATLAB “evalclusters” function (Tibshirani et al., 2001). The muscle weighting components and their corresponding temporal pattern components within the cluster were then averaged (synergy cluster centroids). Next, we sorted the relatively subject‐invariant cluster centroids in muscle weighting components (defined as having synergies from ≥1/3 of the participants) for phase 2 based on the centroids in phase 1 (Cheung et al., 2020; Nazifi et al., 2017). Specifically, the scalar product was computed for every possible pair of synergy cluster centroids in muscle weighting components between parts. Then, we selected the pair with the highest value of similarity, and the synergy cluster centroids involved in that pair were removed from the set. The highest similarity value among the remaining sets was chosen, and the pair was removed until all synergy cluster centroids were appropriately matched. This resulted in similar muscle weighting components, and the corresponding temporal pattern components were of the same order in both parts.

### 3.5 Statistical analysis

A paired‐samples *t*‐test was used to determine the difference in the spatiotemporal variables between the two parts (First vs. Final) using the MATLAB “*t*‐test” function. If the differences between the paired samples were not normally distributed, the Wilcoxon signed‐rank test was applied using the MATLAB “signrank” function. We also compared the number of muscle synergies and the VAF between parts. For the between‐part comparisons of synergy structures, the extracted muscle synergies need to be matched (Lopes Ferreira et al., 2020; Yokoyama et al., 2021). Thus, the comparison was performed if the similarities of each between‐group pair of muscle weighting components defined by the scalar product, and temporal pattern components defined by the max coefficient were >0.8 (Cheung et al., 2020). Specifically, we compared individual weightings of each muscle synergy vector to investigate the differences in the contribution of muscles. This comparison was performed only on muscles that were significantly active. We defined significantly active muscles as those whose 95% confidence interval (CI) did not include zero (Sawers et al., 2015).

Temporal pattern components were compared between parts using statistical parametric mapping (SPM) (spm1d v0.4.7 for MATLAB (Pataky, 2010). The significance level for all tests was set at *p* = 0.05. The *p* values obtained from all tests were corrected using the false discovery rate correction for multiple comparisons (Benjamini et al., 1995). When there was significant difference between the groups, effect sizes (ES) were calculated using Cohen's *d* using the MATLAB “meanEffectSize” function (Cohen, 1988).

We recruited nine subjects without an a priori power analysis, thus, we instead conducted a sensitivity analysis in G*Power, which indicated that an effect size of 0.91 would be necessary to obtain a power of 80% at an *α* of 0.05.

## 4 RESULTS

### 4.1 Spatiotemporal variables during 400‐m

Measured 400‐m time was 50.41 ± 1.66 s (ranged: 48.14–52.91 s). Since subjects performed 400‐m sprints with 14 EMG sensors and datalogger, the measured time was different by about 2–2.4 s from the personal record (48.11 ± 1.6 s) and season best (48.46 ± 2.0 s), respectively.

Table 1 showed the mean and SD of the spatiotemporal variables in the first part and the final part. Running speed, step frequency, and step length were significantly declined in the final part (Running speed: *p* < 0.001, ES = 3.79, Step frequency: *p* < 0.001, ES = 2.43, step length: *p* < 0.001, ES = 3.61). However, contact time was significantly increased in the final part (*p* < 0.001, ES = 4.25), whereas there was no significant difference in flight time between the two parts (*p* = 0.151, ES = 0.47).

**TABLE 1:** Spatiotemporal variables (mean ± SD): Differences between the first part (100–150 m) and the final part (350–400 m).

	First part (100–150 m)		Final part (350–400 m)		Δ (%)		Paired‐T	Effect size
	Mean	SD	Mean	SD	Mean	SD	*p*	*Cohen d*
Spatiotemporal variables								
Running speed (m·s^‐1^)	8.83 ± 0.33		7.04 ± 0.24		79.9 ± 4.0		<0.001*	**4.42** ^a^
Step frequency (Hz)	3.92 ± 0.10		3.55 ± 0.15		90.6 ± 3.5		<0.001*	**2.61** ^a^
Step length (m)	2.25 ± 0.11		1.99 ± 0.09		88.2 ± 2.5		<0.001*	**3.84** ^a^
Contact time (ms)	103.3 ± 7.1		126.3 ± 8.0		121.9 ± 5.4		<0.001*	**4.99** ^a^
Flight time (ms)	139.7 ± 7.2		142.4 ± 13.8		102.0 ± 5.5		0.346	0.33

^a^ Detected effect size (dz > 0.91).

*Significant difference was observed (*p* < 0.001).

### 4.2 Muscle synergies during 400‐m

**FIGURE 1:**
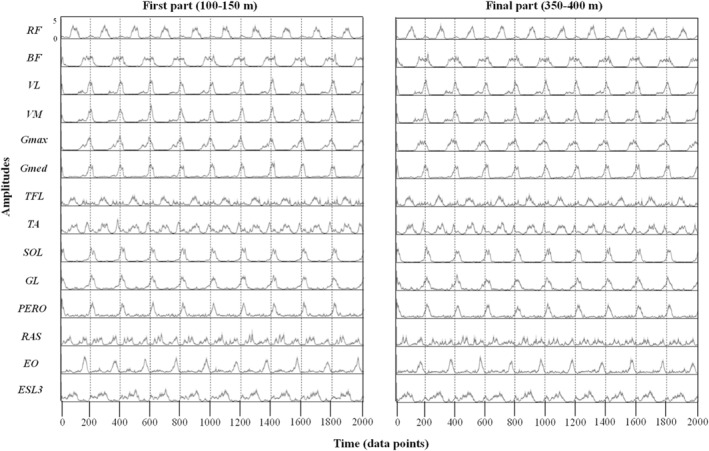
Electromyographic (EMG) signals during the first part (left side) and the final part (right side) from all participants. EMG signals were recorded from 14 muscles of the right side. The mean of the EMG envelopes for all participants are plotted as a line and the standard error as a shading around it. The amplitude is normalized by the maximum value for each muscle over all phases. Vertical dashed lines indicate averaged values of contact timing of the right leg. BF, biceps femoris; ESL3, erector spinae at L3; GMax, gluteus maximus; Gmed, gluteus medius; GL, gastrocnemius lateralis; OE, oblique externus; PERO, peroneus longus; RA, rectus abdominis; RF, rectus femoris; SOL, soleus; TA, tibialis anterior; TFL, tensor fasciae latae; VM, vastus medialis; VL, vastus lateralis.

Figure 1 shows the recorded EMG signals of 14 leg muscles from all participants for each part. Based on visual inspections, there were no clear artifacts in the recorded EMG signals on both parts. Obvious changes between parts in the EMG patterns based on visual inspection were not identical. The VAF for each phase was presented in the Figure S1. The extracted number of synergies was 7.0 ± 0.7 (mean ± SD) for the first part 1 and 7.2 ± 0.4 for the final part. There were no significant differences in the number of synergies between parts (*p* = 0.69).

**FIGURE 2:**
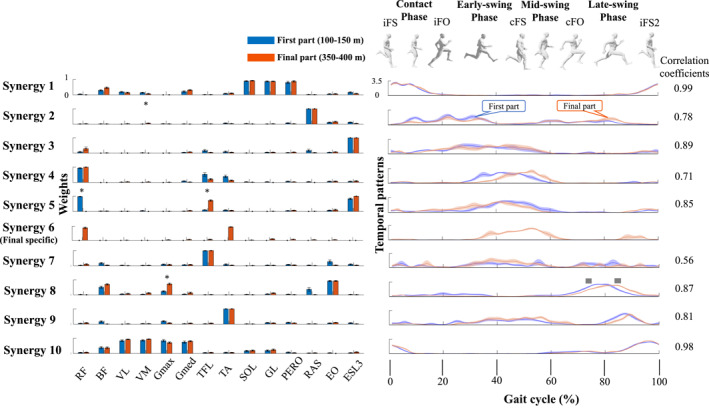
The muscle weighting components vectors are shown on the left side of the figure aligned to the corresponding temporal pattern component. Nine synergy clusters were identified in the first part and ten in the final part, with Synergy 6 being specific to the final part. Each colored bar representing weighting components and each colored waveform indicates temporal patterns corresponding to the first (blue) and final (orange) parts of the sprinting sequence. The asterisk (*) denotes significant differences in the individual weightings of each muscle synergy vector and in the temporal patterns between the two parts. The right side of the figure displays the correlation coefficients for temporal patterns between these two parts. The vertical dashed lines indicate averaged values of foot‐off timing of ipsilateral leg (iFO), foot‐strike timing of contralateral leg (cFS), and foot‐off timing of contralateral leg (cFO) of first (blue) and final (orange). Note that foot‐strike timing of the ipsilateral leg (iFS) is 0% of the gait cycle. The gray sections on the *X*‐axis of temporal patterns represent the temporal pattern components where a significant difference between running and functional tasks was observed (*p* < 0.05). BF, biceps femoris; ESL3, erector spinae at L3; GL, gastrocnemius lateralis; Gmax, gluteus maximus; Gmed, gluteus medius; OE, oblique externus; PERO, peroneus longus; RA, rectus abdominis; RF, rectus femoris; SOL, soleus; TA, tibialis anterior; TFL, tensor fasciae latae; VM, vastus medialis; VL, vastus lateralis.

Figure 2 shows the cluster centroids of muscle weighting components and the cluster centroids of the temporal pattern components in both parts. The cluster analysis identified nine clusters in the first part 1 and ten clusters in the final part. Table 2 shows the contributions of muscle activations and activations parts based on visual inspections of muscle weighting components, and temporal pattern components, respectively.

**TABLE 2:** The contributions of muscle activations and activations phases based on visual inspections of muscle weighting components, and temporal pattern components.

	Muscle weighting (major contributions)	Temporal patterns (phase)
Synergy 1	SOL, GL, PERO	Contact
		Late‐swing
Synergy 2	RAS	Contact
		Early‐swing
Synergy 3	ESL3	Contact
		Early‐swing
		Mid‐swing
Synergy 4	RF, TFL, TA	Early‐swing
		Mid‐swing
Synergy 5	RF, ESL3	Early‐swing
		Mid‐swing
Synergy 6 (final‐specific)	RF, TA	Early‐swing
		Mid‐swing
Synergy 7	TFL	Early‐swing
		Mid‐swing
Synergy 8	BF, Gmax, EO	Late‐swing
Synergy 9	TA	Late‐swing
Synergy 10	VL, VM, Gmax and Gmed	Late‐swing
		Contact

Abbreviations: BF, biceps femoris; ESL3, erector spinae at L3; GL, gastrocnemius lateralis; Gmax, gluteus maximus; Gmed, gluteus medius; OE, oblique externus; PERO, peroneus longus; RA, rectus abdominis; RF, rectus femoris; SOL, soleus; TA, tibialis anterior; TFL, tensor fasciae latae; VL, vastus lateralis; VM, vastus medialis.

For between‐group comparison, the muscle weighting component of synergy 2 in the VM was significantly larger for the final part than for those in the first part (*p* = 0.038, ES = 1.12) (Figure 2). The muscle weighting component of synergy 5 in the RF was significantly larger for the first part than for those in the final part (*p* = 0.016, ES = 47.38), and the TFL was significantly larger in the final part than for the first part (*p* = 0.016, ES = 4.73). The muscle weighting component of synergy 8 for the Gmax was larger for the final part than for the first part (*p* = 0.003, ES = 2.55). The SPM analysis revealed a significant difference in the temporal pattern components of synergy 8 between the first and final parts. Specifically, there was a significantly larger temporal pattern in the late‐swing phase of the first part (73.7%–75.2% of the gait cycle) (*p* = 0.027, ES = 1.57). By contrast, there was a significantly lower temporal pattern in the late‐swing phase of the final part (84.6%–86.4% of the gait cycle) (*p* = 0.022, ES = 2.35).

## 5 DISCUSSION

The hypothesis of this study was that disruptions in the neuromuscular coordination of multiple muscles, especially thigh muscle, would be observed with performance decline for the 400‐m sprints. In this study, we applied the NMF algorithm to the EMG data of the first (100–150 m) and final part (350–400 m) of 400‐m sprint to investigate the changes of lower‐limb muscle synergies in the presence of declined performances induced by fatigue. Overall, the number of muscle synergies did not change between the two parts of 400‐m sprint, but we identified a specific muscle synergy, and alterations in the individual muscle weightings of and temporal patterns of several hip and thigh muscles specifically in the final part. However, no change in the muscle weighting of shank muscles and temporal patterns of all muscles even following fatigue in the 400‐m sprint. Thus, our hypothesis was accepted.

Sprinters typically declined their running speed, step frequency, and length, while experiencing increased contact time during the final part of the 400‐m sprint. These observations, quantified by the effect sizes detailed in Table 1, align with previous studies (Hanon et al., 2009). We suggest that the declined step frequency and length correlate with the altered muscle synergy patterns observed in our study. Primarily, the majority of muscle synergies demonstrating significant differences between the first and final parts were related to hip muscles during the early to late‐swing phases. These shifts in muscle weighting in response to fatigue agree with a prior study (Hajiloo et al., 2020).

The RF, a principal hip flexor, plays a critical role in high‐speed running (Dorn et al., 2012; Kakehata et al., 2023; Schache et al., 2015). Synergies 4 and 5 in the first part exhibit larger RF activation during the early swing phase, suggesting they were the primary synergies for hip flexor production necessary in the sprint. In synergy 4, RF activation occurred synchronously with TFL, a hip flexor, and TA, responsible for ankle dorsiflexion, facilitating hip flexion and foot lift‐off. In synergy 5 of the first part, the activation of RF was accompanied by the activation of ESL3, which may counteract the spinal flexion force produced by the RF through the pelvis, thus maintaining an upright posture. However, due to its significant role in sprinting, the RF is prone to fatigue as a consequence of repetitive lower‐limb action (Brocherie et al., 2015; Girard et al., 2020). The significantly lower RF activation in synergy 5 during the final parts suggests that sprinters could not maintain high RF activation in the early swing phase to recover the leg. This resulted in an increase in TFL activation in synergy 5 as a compensation strategy for hip flexion due to the lower activation of RF in Synergy 5. However, the formation of final‐part specific synergy (Synergy 6) with RF and TA activations may suggest that the sprinters presented additional RF and TA activations during the early to mid‐swing phase in the final part to support swing movement, which was also found in the deceleration phase of 100‐m dash (Kakehata et al., 2022).

In the mid to late swing phase, we observed heightened activation of the Gmax in synergy 8 during the final part. While the precise mechanism behind this remains complex, it might stem from the overactivation of TFL in synergy 5 during the final part, resulting in excessive internal rotation of the hip joint. Therefore, in the final part, sprinters needed to enhance Gmax activation, the external hip rotator, to counteract this internal rotation force and stabilize the hip joint. Moreover, previous research suggests that Gmax activation contributes to increased step frequency during high‐speed running (Dorn et al., 2012). It also plays a crucial role in coordinating rapid hip extension with hamstring muscles, such as BF, to produce horizontal ground reaction forces (Morin et al., 2015). These forces are a critical factor in determining step length (Hunter et al., 2005). In the final part of the sprint, the altered muscle synergies induced by fatigue seem to prompt a compensatory strategy. This strategy involves a more substantial activation of the Gmax muscles, aiming to increase the contribution of hip extension in the late swing phase, thereby attempting to minimize the decline in step length and speed. Interestingly, we observed a significantly lower temporal pattern at the start of the late‐swing phase in the final part, which increased towards the end of the late‐swing phase in the final part when compared with those in the first part. This suggests that the sprinter increased the effort to activate Gmax toward the end of the swing phase to sustain hip extension. The strategy to minimize the decline in step length toward the final part of the 400‐m sprint can also be observed in the knee joint during the latter half of the contact phase. We noted a significant increase in the muscle activation of VM in the final part of synergy 2 (see Figure 2). Although its activation level was relatively small, the increased VM activation may contribute to pushing more forcefully against the ground through the knee joint, in conjunction with the plantar flexor patterns of synergy 1, in the final part. This observation aligns with a previous study showing that the contact leg's knee joint angle during the push‐off phase was in a more extended position at the 380‐m mark than at the 125‐m mark during a 400‐m sprint (Saraslanidis et al., 2011).

Lastly, we found no significant difference in the individual muscle weightings and the temporal pattern components of plantar flexors (i.e., SOL, GL, and PORO) in Synergy 1 between the first and final parts. This contrasts with previous studies showing decreased plantar flexor activations in response to fatigue (Billaut et al., 2009; Girard et al., 2020). The discrepancy could potentially be attributed to different study protocols. Earlier studies typically employed more specific, intense fatigue protocols, often consisting of short durations and multiple sessions. For instance, a study that reported altered activations in plantar flexor muscles post‐fatigue protocol (Girard et al., 2020), incorporated three sets of 5‐s maximal sprints, each separated by 2 min of passive rest. Another study that observed altered temporal patterns (Smale et al., 2016) applied fatigue protocols involving non‐running tasks, such as repetitive squats and maximum isometric contractions. These specific fatigue protocols likely led to fatigue in multiple muscles, including those in the hip and lower leg, rather than just the hip muscles as observed in the 400‐m sprints in our study. It's plausible that we may observe fatigue in both thigh and shank muscles if athletes engage in high‐speed running over distances exceeding 400‐m. Secondly, previous research often overlooked the inclusion of sprinters who had undergone extensive periods of training (Billaut et al., 2009; Girard et al., 2020; Hajiloo et al., 2020). As such, it's conceivable that the sprinters participating in our study could execute sprints optimized for speed endurance ability. This might be achieved by minimizing changes in individual muscle weightings (i.e., alterations primarily observed in the hip muscles during the final part) and maintaining consistent temporal patterns, likely benefitting from long‐term motor training. In this study, we recruited only 400‐m sprinters, however, further study is needed to clarify the group differences of muscle synergy and its relation to the running performances among various athletic levels in the future. In conclusion, current findings suggest that notable changes of hip and thigh muscle activations would be one of the factors in the decline of the 400‐m sprint.

## 6 CONCLUSION

This study reveals that fatigue‐induced performance decline in a 400‐m sprint corresponds with alterations in muscle synergies, particularly in the hip and thigh muscles. There were notable shifts in RF activation timings and an increased activation of TFL and Gmax as compensatory strategies. These insights highlight the need for training concepts that manage muscle coordination patterns and improve speed endurance ability to optimize sprinting performance.

## CONFLICT OF INTEREST STATEMENT

There is no conflict of Interest in this study.

## Supporting information

Supporting Information S1
