# Supplementary material for: Changes in muscle coordination patterns during 400‐m sprint: Impact of fatigue and performance decline
Source: Eur J Sport Sci. 2024 Feb 17;24(3):341–51. doi: 10.1002/ejsc.12085 (PMC11236024; doi:10.1002/ejsc.12085)
Supplement: Supplementary file 1 — Supporting Information S1 [file EJSC-24-341-s001.docx]

**Supplementary Figure 1: The variability accounted for (VAF) for first (blue) and final part (red).**
